# Experimental Phaeohyphomycosis of *Curvularia lunata*

**DOI:** 10.3390/jcm11185393

**Published:** 2022-09-14

**Authors:** Najwa Al-Odaini, Kai-su Pan, Liu-wei Liao, Nan-fang Mo, Zhi-wen Jiang, Tian-tian Li, Xiu-ying Li, Xiao-juan He, Dong-yan Zheng, Cun-wei Cao

**Affiliations:** 1 Department of Dermatology and Venereology, The First Affiliated Hospital of Guangxi Medical University, Nanning 530021, China; 2 Guangxi Health Commission Key Lab of Fungi and Mycosis Research and Prevention, Nanning 530021, China

**Keywords:** *Curvularia lunata*, phaeohyphomycosis, in vitro drug susceptibility testing, animal model, dematiaceous fungi

## Abstract

Originally considered to be a plant pathogen, reports of phaeohyphomycosis due to *Curvularia lunata* (*C. lunata*) in animals and humans are increasing. However, studies on the pathogenesis, virulence, and epidemiology of *C. lunata* have rarely been discussed. In the present study, BALB/c mice were experimentally inoculated with *C. lunata* suspension by different routes and the course of infection was evaluated. In addition, the in vitro antifungal susceptibility of *C. lunata* against six commonly used antifungals was evaluated using the microdilution method. Inoculation resulted in skin lesions in animals inoculated intraperitonially and subcutaneously. Infection was confirmed by both mycological and histopathologic examination. *C. lunata* spores and hyphae were detected in the histopathologic sections stained with hexamine silver staining. In addition, voriconazole (VRC) demonstrated greater activity against *C. lunata* when compared to the other antifungals, whereas fluconazole (FLC) was the least active antifungal with a minimum inhibitory concentration (MIC) range of 8–16 μg/mL. Further studies are necessary to understand the pathogenicity of *C. lunata* and uncover the mystery of this fungus.

## 1. Introduction

Phaeohyphomycosis is a group of infections caused by dematiaceous, darkly pigmented fungi, the dark pigmentation is due to the presence of melanin in the cell wall of the fungi, which plays an important role in the virulence and pathogenesis of these fungi [1,2]. Dematiaceous fungi are abundant in the environment, and with the widespread use of antibiotics, immunosuppressants, and corticosteroids, a broad and expanding list of fungi causing pheohyphomycosis has been reported, causing a variety of clinical syndromes in humans and animals, including *Cladophialophora*, *Curvularia*, *Rhinocladiella*, *Verruconis*, *Exophiala*, and *Fonsecaea* [3]. *C. lunata* is the most common species of the genus *Curvularia* formerly causing diseases confined to plants and animals. However, in the last two decades, infections in humans caused by *C. lunata* have increased significantly. These infections include allergic sinusitis, keratitis, cutaneous and subcutaneous infections, pulmonary infections, central nervous system infections, peritonitis, and disseminated diseases [4,5,6,7,8,9,10,11,12,13,14,15,16,17,18,19]. Despite this, studies on *C. lunata* in experimental phaeohyphomycosis and the pathogenesis of this emerging pathogen are very limited. The mainstay of treatment for *C. lunata* infection is antifungal therapy and surgery when needed. Different classes of antifungals were used in the literature, nevertheless, the optimal antifungal for this infection remains to be determined. We recently reported a novel case of tinea nigra in an immunocompetent child where *C. lunata* was isolated from the skin lesion [20]. Thus, the main objective of the current study was to investigate the possibility of producing a tinea nigra lesion by experimenting on an animal model, in addition to evaluating the in vitro activity of commonly used antifungals against *C. lunata*.

## 2. Methods

### 2.1. Curvularia Lunata Isolates

Two clinical isolates of *C. lunata* (CL2021 and CL2022), previously identified by morphological examination and ITS sequence analysis at the Guangxi Health Commission Key Lab of Fungi and Mycosis Research and Prevention were obtained and included in the study. 

### 2.2. Animals

Twenty-six white male BALB/c mice, aged 8–10 weeks, and weighing approximately 25–30 g were obtained from Charles River Co, Ltd., Beijing, China, and housed in a biosafety level 3 experimental facility according to the requirements for experimental animals. All experimental procedures and animal care were performed according to protocols approved by the Ethics Committee of the First Affiliated Hospital of Guangxi Medical University (No. 2019KY-E-048). The experiment was performed as described [21] with minor modifications. Briefly, mice were randomly divided into three groups according to the route of inoculation (transdermal, subcutaneous and intraperitoneal) in addition to one control group (*n* = 2). After inoculation, the mice were observed daily for 1 to 7 weeks. One mouse from each group was sacrificed after two, four, and six weeks, respectively, and were all examined macroscopically for lesions on the liver, spleen, lungs, heart, and kidneys. Specimen from organs with or without gross lesions were cultured on Sabouraud Dextrose Agar (SDA) medium for fungal detection. Mice with skin lesions were subjected to skin biopsies.

### 2.3. Preparation of Inoculum

Suspensions used for inoculation were prepared from *C. lunata* colonies cultivated on SDA medium at 25 °C for 7 to 14 days. After complete growth, the surface of the colonies was washed and gently scraped from the medium and placed in a tube containing normal saline to obtain a fungal suspension with a final concentration of 1 × 10^9^ CFU/mL for later use.

### 2.4. Inoculation

#### 2.4.1. Transdermal Inoculation

Eight mice were randomly divided into two groups: (i) normal immunity group (*n* = 4), (ii) immunocompromised group (*n* = 4). The immunocompromised group received an intraperitoneal injection of 200 mg/kg cyclophosphamide (MACKLIN Co., Ltd., Shanghai, China) 4 days before the experiment, 1 day before, and on the day of inoculation. After anesthetization with chloral hydrate (Krohne Co., Ltd., Chengdu, China) (0.25 mL/mouse intraperitoneally), the dorsal aspects of the mice were shaved with a razor blade, disinfected with 75% ethanol, and the exposed area was gently abraded with a sterile sandpaper until glistening. Following this, a 0.2 mm sterilized derma-roller was used to pierce channels in the prepared area to facilitate penetration of the fungal suspension. The site was then disinfected with 75% ethanol, and after drying, a suspension of 100 µL was applied and lightly rubbed into the abraded area with a sterile blade. Finally, the infected area was covered with a sterile transparent film dressing (Minnesota Mining Manufacturing Medical Equipment Co., Ltd., Shanghai, China).

#### 2.4.2. Subcutaneous Inoculation

Ten mice were randomly divided into three groups according to the site of inoculation: (i) dorsal aspect (*n* = 2); (ii) forepaws (*n* = 4); (iii) hind paws (*n* = 4). The immunity of the two mice inoculated on the dorsal aspect was reduced with 200 mg/kg cyclophosphamide at the time of inoculation. All mice were injected subcutaneously with 0.2 mL of the suspension. The contralateral side of the inoculated hind paws or forepaws served as control.

#### 2.4.3. Intraperitonially Inoculation

Six mice were randomly divided into four immunosuppressed mice (200 mg/kg cyclophosphamide at the time of inoculation) and two mice with normal immunity. The dorsal and ventral aspects of the mice were shaved with a razor blade, disinfected with 75% ethanol. Following, each mouse was injected intraperitoneally with 0.2 mL of the fungal suspension.

### 2.5. Clinical Evaluation

A successful skin infection was established if one or more of the following conditions were present: (1) papules, (2) hyperpigmented spots, (3) scales, (4) crusts, (5) ulcers, (6) nodules, (7) hyphae and spores could be seen in direct microscopic examination of skin lesions, (8) positive fungal cultured from skin lesions, (9) fungal spores of hyphae were seen in the histopathological examination of skin lesions.

### 2.6. In Vitro Antifungal Susceptibility 

Antifungal susceptibility was tested using the checkerboard broth microdilution method outlined in CLSI document M38-A2 [22]. A suspension containing *C. lunata* isolates was standardized to a final optical density of 1 × 10^3^–5 × 10^3^ CFU/mL using a spectrophotometer at 530 nm. The drugs used were all original powders (Sigma, St. Louis, MO) with a purity of ≥99%. Fluconazole was dissolved with sterile distilled water, while sterile dimethyl sulfoxide (DMSO) solvent was used to dissolve the lipid-soluble drugs: amphotericin B (AMB), itraconazole (ITC), terbinafine (TRB), ketoconazole (KCZ) and VRC. The final drug concentrations ranges of each drug were: 0.125–64 μg/mL for FLC and KCZ, 0.0015–8 μg/mL for AMB and ITC, 0.0020–1 μg/mL for VRC, and 0.0031–16 μg/mL for TRB. The solutions were then diluted in RPMI 1640 medium and the microplates containing the antifungal solutions and each fungal suspension were incubated at 35 °C, and assays were read after 48 hours. The MIC was defined as the lowest concentration of antifungal capable of inhibiting 100% (AMB), 50% (ITC, VRC, FLC, and KCZ), and 80% TRB of fungal growth compared to drug-free growth control [23,24]. *Candida parapsilosis* ATCC22019 and ATCC6458 strains were used for quality control. The susceptibility test was performed in triplicate to obtain optimal results.

## 3. Results

**Figure 1:**
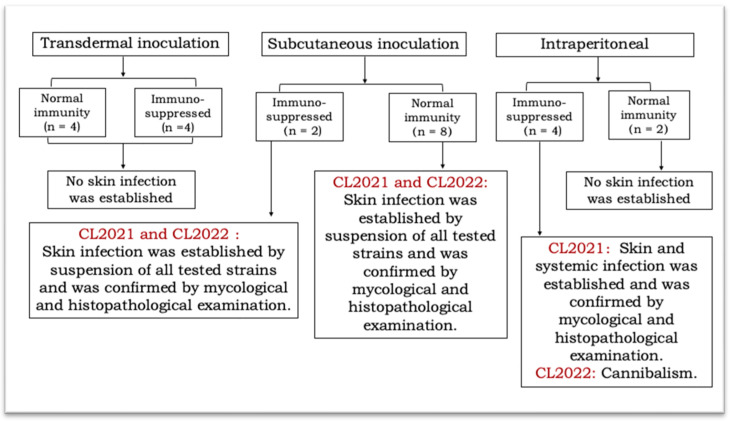
Schematic of experimental procedure and results.

Schematic of experimental procedure and results are shown in Figure 1.

### 3.1. Transdermal Inoculation

Erythema, mild ulceration, and scaling were observed on the skin of infected mice. However, no visible infection was established in this group and skin scraps for mycological examination were negative. In addition, the fungus was not reisolated from the culture of the skin sample from the inoculation site or from the internal organs.

### 3.2. Subcutaneous Inoculation

**Figure 2:**
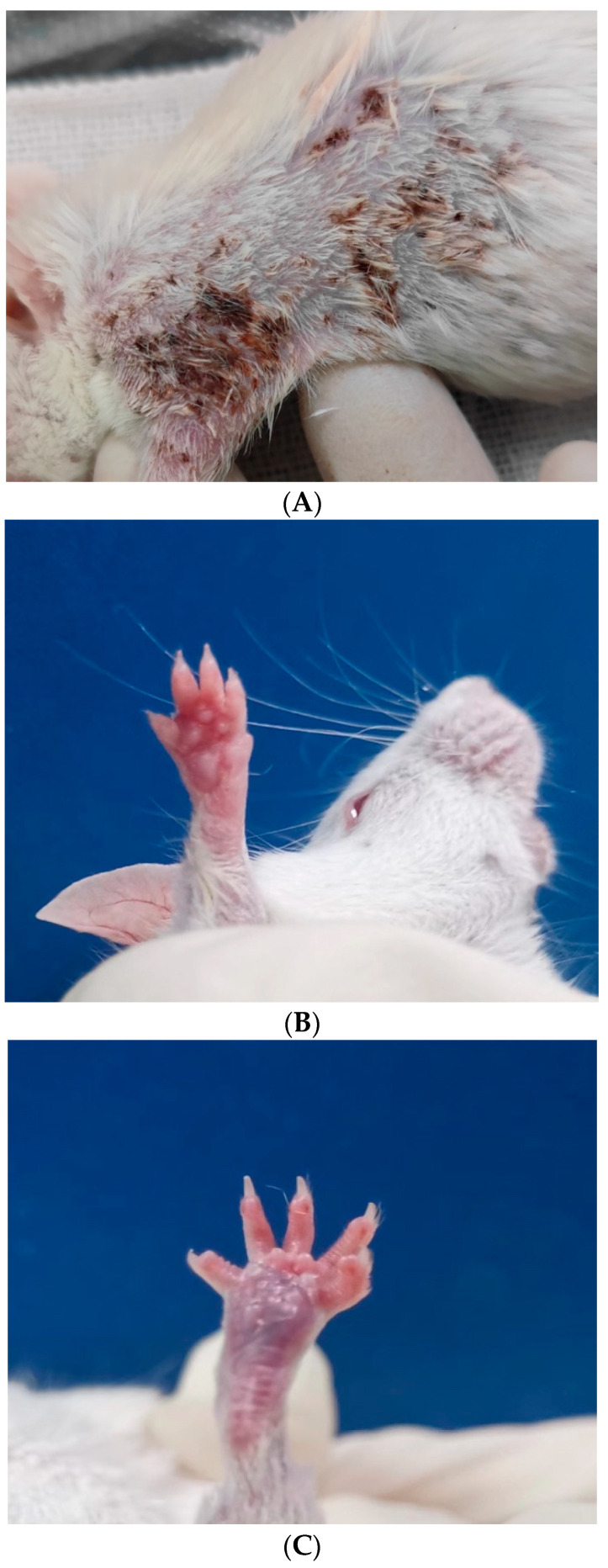
Subcutaneous inoculation (**A**) Bleeding spots, hyperpigmentation and scales gradually appeared on the skin of the dorsal aspect of the mouse [14 days after inoculation]; (**B**) Redness and swollen forepaw [7 days after inoculation]; (**C**) 24 days after inoculation, obvious signs of inflammation (swelling and congestion) appeared on the hind paw of infected mouse.

**Figure 3:**
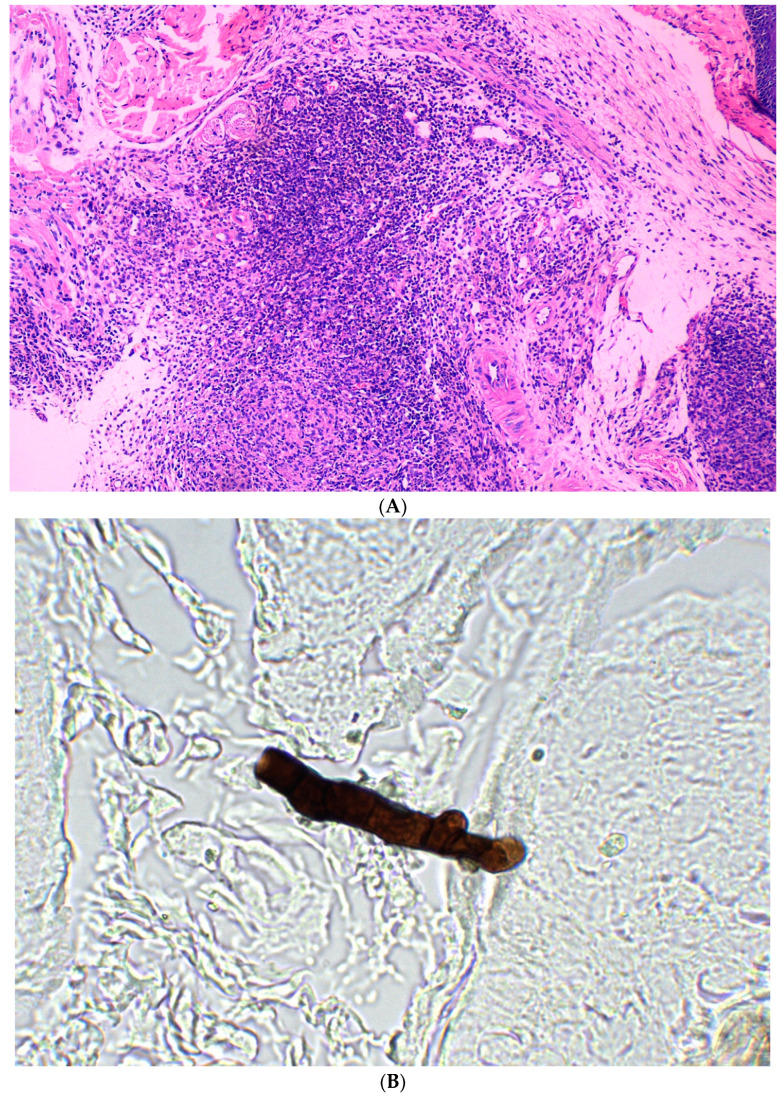
Histopathology of skin specimen (**A**) severe inflammatory cells infiltration in the dermis after subcutaneous inoculation [forepaw] (HE ×100); (**B**) dark hyphae in the dermis [forepaw] (hexamine silver staining × 1000).

Infection was established in all 10 mice inoculated subcutaneously. The first signs of infection were observed on the skin of the ventral aspect of the infected mice on day 7 after inoculation and manifested as bleeding spots, hyperpigmentation, and scaling. The lesions gradually increased in size and were covered with brown-black crusts. Obvious signs of infection manifested as swelling, congestion, and slight scaling appeared on the infected forepaws and hind paws (Figure 2). *C. lunata* could be reisolated from skin and epidermal scrapings of all inoculated animals from day 7 onward. Histological sections of skin and subcutaneous tissue stained with hematoxylin-eosin (HE) showed inflammatory cell infiltration. Spores and hyphae of *C. lunata* were detected in the dermis in the histological sections stained with hexamine silver (Figure 3). *C. lunata* could not be reisolated from specimens obtained from internal organs of the infected mice.

### 3.3. Intraperitoneal Inoculation

**Figure 4:**
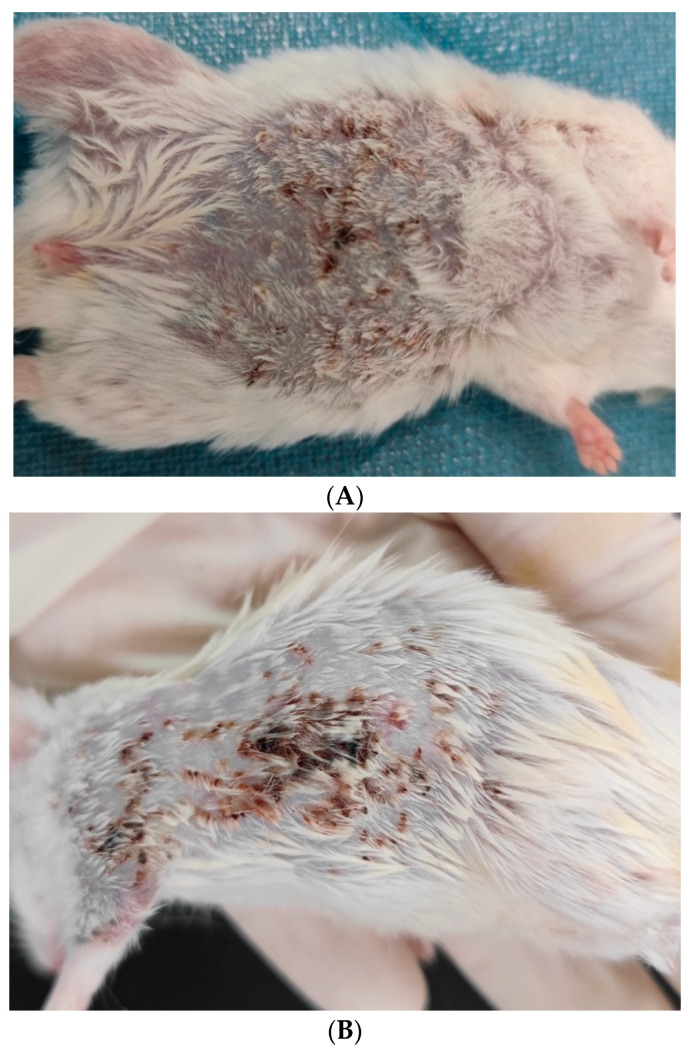
Intraperitoneal inoculation: (**A**) bleeding spots, hyperpigmentation and scales gradually appeared on the skin of the ventral aspect of the mouse 7 days after inoculation; (**B**) dorsal aspect of the mouse 24 days after inoculation.

**Figure 5:**
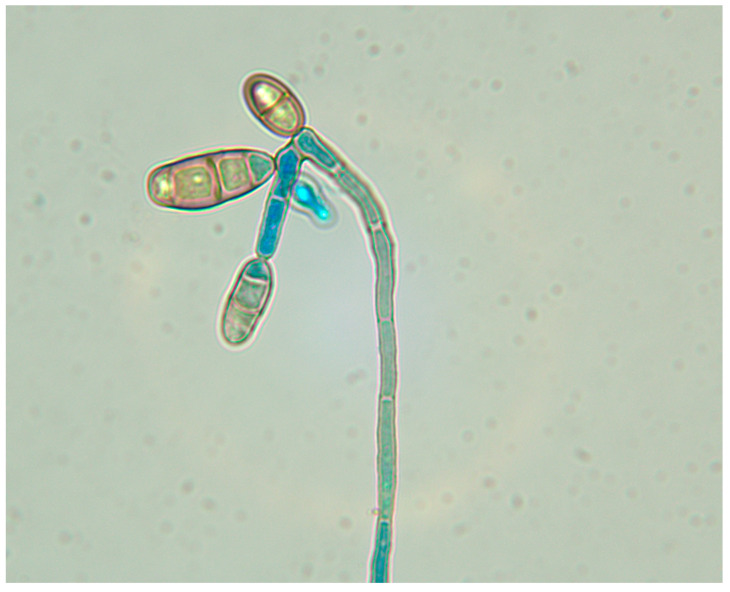
Characteristic morphology of the *C. lunata* isolated from infected samples cultured on SDA at 25 °C.: curved conidia with swollen subterminal cell (lactophenol cotton blue mounting medium].

**Figure 6:**
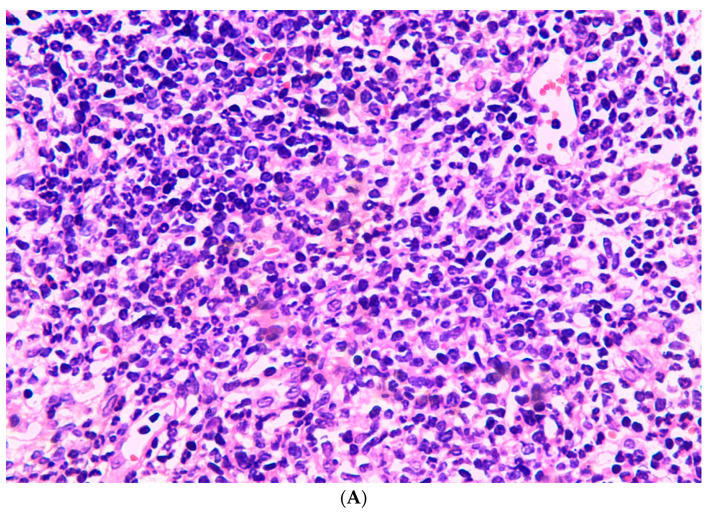

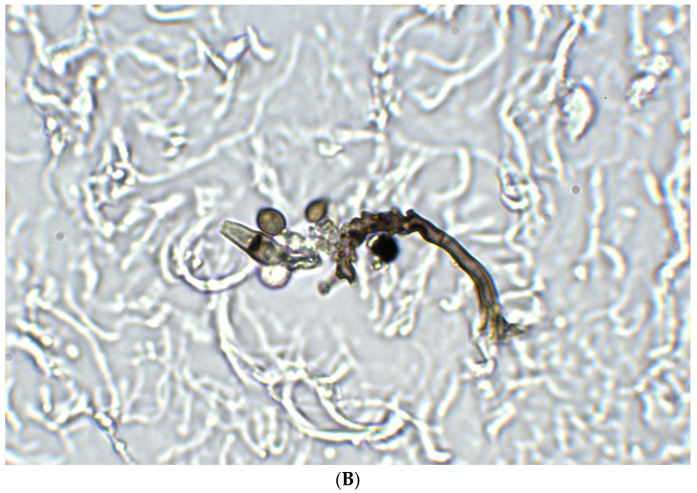
Histopathology of skin specimen (**A**) inflammatory cells infiltration in the dermis after intraperitoneal inoculation [dorsal aspect] (HE × 400); (**B**) dark hyphae and spores in the dermis [dorsal aspect] (hexamine silver staining × 1000).

**Figure 7:**
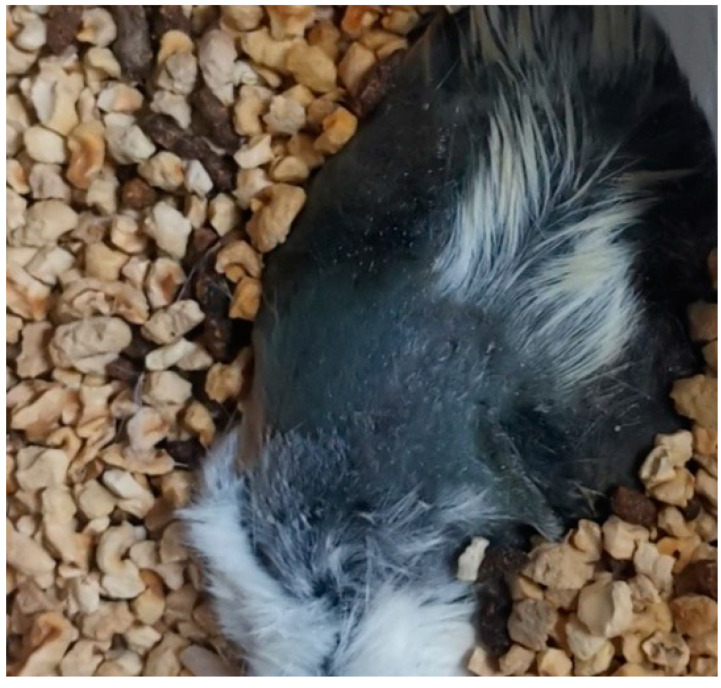
Widespread dark discoloration of the skin of the deceased mouse due to cannibalism.

Intraperitoneal inoculation resulted in systemic infection in two immunosuppressant-treated mice. Skin lesions appeared 7–12 days after intraperitoneal inoculation, manifested as bleeding spots that gradually increased in number and size and developed into erosions and crusts (Figure 4). The macroscopic appearance of internal organs showed no abnormalities; however, *C. lunata* was reisolated from specimens obtained from skin, liver, kidney, spleen, and lung cultured on SDA medium (Figure 5). Histopathologic examination showed severe inflammatory cell infiltration with the presence of hyphae in the dermis (Figure 6). The remaining immunosuppressant-treated mice showed aggressive behavior during the observation period and were found deceased. The cause of death was cannibalism, which was confirmed by autopsy (Figure 7). *C. lunata* was reisolated from skin specimens obtained from the deceased mouse and cultured on SDA. No infection was detected in the group with normal immunity.

### 3.4. In Vitro Antifungal Susceptibility

Currently, there are no available CLSI criteria for *C. lunata* susceptibility, therefore, the results are reported without interpretation. Strains of *C. lunata* were most susceptible to VRC with a MIC range of 0.25–0.5 μg/mL. Terbinafine and AMB also had good antifungal activity against *C. lunata* with MIC values of 1 μg/m and 2 μg/mL, respectively. Itraconazole and KCZ were also effective, with MIC values of 2–4 μg/mL and 1–2 μg/mL, respectively. Fluconazole was the least active antifungal agent against *C. lunata*, with MIC values of 8–16 μg/mL (Table 1).

## 4. Discussion 

*C. lunata* in experimental pheohyphomycosis has been reported on a few occasions in the literature, most of which involved keratitis. According to previous reports, the skin lesions of *C. lunata* infection may be single or multiple dark brown or red nodules with an eschar in the center that sometimes develops into an abscess [19,25,26,27]. Tinea nigra caused by *C. lunata* prompted us to conduct the current study. In the present study, infection was established with a suspension of *C. lunata* isolated from clinical specimens and subsequently cultured in the laboratory. Our first attempt was to produce a tinea nigra-like lesion, with consideration to abrasion and occlusion of the inoculation site, as described by Ritchie EB et al. [28], where he successfully produced a tinea nigra lesion with a suspension of the filamentous phase of *Hortaea werneckii* into a bleeding scarified area of a human volunteer by transdermal inoculation. However, although various solutions were used to prepare the suspension, including distilled water, saline, and sucrose, as well as various abrasion methods, we were unable to produce skin infection by transdermal inoculation of *C. lunata*. We suspect that this may be due to differences between the skin structure of humans and mice or due to the short observation time. In contrast to Whitcomb et al. [21], *C. lunata* caused skin lesions and was reisolated from subcutaneously inoculated animals. It is noteworthy that disseminated infections due to *C. lunata* infection in the literature were mostly caused by cutaneous infections and not vice versa. Rout N et al. [29], reported that inoculation of a spore suspension of *C. lunata* in albino rats resulted in a localized lesion and simultaneous steroid therapy resulted in lesion in distant organs. Interestingly, we detected skin lesions in the immunocompromised mice seven days after intraperitoneal inoculation, which progressively increased in numbers with increasing observation time, and cultures of the skin lesions and internal organs yielded *C. lunata*. Although we were unable to establish infection in the group with normal immunity by similar route, skin infection was established in mice with normal immunity that were inoculated subcutaneously into the forepaws and hind paws. In this context, *C. lunata* infection has been sporadically reported in immunocompetent individuals [30,31], suggesting that *C. lunata* infection is possibly irrespective of the host immune status if circumstances are favorable.

In addition, aggressive behavior and death due to cannibalism were noted in our experimental animals. Unfortunately, we were unable to obtain a specimen to confirm central nervous system involvement, but interestingly, widespread dark discoloration was observed in the skin of the deceased mouse and *C. lunata* was reisolated from the culture of the skin specimens. This could be due to the accumulation of melanized fungi in the deceased animal. 

Currently, there are no defined clinical breakpoints or epidemiologic cutoff values for *C. lunata*. Therefore, in vitro drug susceptibility testing is of great importance to guide treatment, which should be individualized according to the susceptibility results. The current study showed that FLC was the least active antifungal drug against *C. lunata* with GM = 10.4 μg/mL, followed by ITC (GM = 2.8 μg/mL), AMB (GM = 2 μg/mL), TRB (GM = 1 μg/mL), and KCZ (1.4 ng/mL), while VRC was the most effective drug (GM = 0.35 μg/mL). Recent studies have shown that isolates of *Curvularia* spp. have satisfactory sensitivity to AMB with a MIC of <2 μg/mL [32,33,34,35]; however, contradictory results have also been reported (MIC values 4–16 µg/mL) [23]. Both *C. lunata* isolates in our study showed susceptibility to amphotericin B with an MIC of 2 µg/mL. Furthermore, *C. lunata* not susceptible to FLC with high MIC values (≥16 µg/mL) has been reported previously [34,35,36]. In contrast, we determined MIC values of ≤16 µg/mL; however, such values should draw attention to the wide use of FLC in clinical practice which may lead to the development of resistant strains. Moreover, VRC showed high antifungal activity against all tested strains, with MIC values of ≤0.5 µg/mL, which is lower than previously reported values [23,32]. Itraconazole, on the other hand, showed high efficacy against *C. lunata* in a previous study (MIC range 0.125–1 μg/mL) [35], in contrast with that of our results with MIC ranges of MIC 2–4 µg/mL.

Nizam et al. [37] and Krizsán et al. [38] described an MIC range of 4 μg/mL and 0.25-8 μg/mL, respectively, for TRB against the genus *Curvularia*. Our study showed that TRB was effective against all tested isolates with a MIC of 1 μg/mL. Moreover, the tinea nigra lesion caused by *C. lunata* was successfully treated with topical KCZ, and the strains tested showed an MIC range of 1–2 µg/mL, which is considered susceptible compared to higher values reported in the literature (≤4 µg/mL) [34].

The results of the in vitro antifungal susceptibility test and the clinical symptoms of the cases reported in the literature suggest that there is little intraspecific difference in the pathogenicity of *C. lunata*. Therefore, early identification of the fungal species has important guiding significance for clinical management. 

Our study has limitations worth mentioning. One is the small sample size. Second, the short observation period and the type of animal could have affected the outcome of the experiment. Future research with a larger sample size and longer observation period on other animal models is needed to further investigate the pathogenicity of *C. lunata*.

In conclusion, we speculate that *C. lunata* is an opportunistic pathogenic fungus that can cause infection irrespective of the host immune status when favorable circumstances exist. These favorable circumstances remain to be explored. Another debated question is the optimal antifungal therapy for *C. lunata* infection, which remains to be determined. The understanding of the pathogenicity of *C. lunata* is still far from satisfactory, and the time to address this emerging fungus is now.

## Figures and Tables

**Table 1:** In vitro antifungal susceptibility of *C. lunata.*

Isolates and Drugs	MIC Range	GM
	(μg/mL)	(μg/mL)
*C. lunata* (*n* = 2)		
FLC	8–16	11.3
AMB	2	2
ITC	2–4	2.8
VRC	0.25–0.5	0.35
TRB	1	1
KCZ	1–2	1.4

MIC: Minimum inhibitory concentration. GM: Geometric Mean. AMB: Amphotericin B. FLC: Fluconazole. ITC: Itraconazole. VRC: Voriconazole. TRB: Terbinafine. KCZ: Ketoconazole.

## Data Availability

Not applicable.
